# Development of a prognostic model based on demographic, environmental and lifestyle information for predicting incidences of symptomatic respiratory or gastrointestinal infection in adult office workers

**DOI:** 10.1186/s13063-016-1668-7

**Published:** 2016-11-16

**Authors:** Tapani Hovi, Jukka Ollgren, Jaason Haapakoski, Carita Savolainen-Kopra

**Affiliations:** National Institute for Health and Welfare, PO Box 30, 00271 Helsinki, Finland

**Keywords:** Prediction model, Acute respiratory infection, Acute gastrointestinal infection, Risk factor, Fixed variable, Age, Gender, Public transport, Chronic disease

## Abstract

**Background:**

Occurrence of respiratory tract infection (RTI) or gastrointestinal tract infection (GTI) is known to vary between individuals and may be a confounding factor in the analysis of the results of intervention trials. We aimed at developing a prognostic model for predicting individual incidences of RTI and GTI on the basis of data collected in a hand-hygiene intervention trial among adult office workers, and comprising a prior-to-onset questionnaire on potential infection-risk factors and weekly electronic follow-up reports on occurrence of symptoms of, and on exposures to RTI or GTI.

**Methods:**

A mixed-effect negative binomial regression model was used to calculate a predictor-specific incidence rate ratio for each questionnaire variable and for each of the four endpoints, and predicted individual incidences for symptoms of and exposures to RTI and GTI. In the fitting test these were then compared with the observed incidences.

**Results:**

Out of 1270 eligible employees of six enterprises, 683 volunteered to participate in the trial. Ninety-two additional participants were recruited during the follow-up. Out of the 775 registered participants, 717 returned the questionnaire with data on potential predictor variables and follow-up reports for determination of outcomes. Age and gender were the strongest predictors of both exposure to, and symptoms of RTI or GTI, although no gender difference was seen in the RTI incidence. In addition, regular use of public transport, and history of seasonal influenza vaccination increased the risk of RTI. The individual incidence values predicted by the model showed moderate correlation with those observed in each of the four categories. According to the Cox-Snell multivariate formula the model explained 11.2% of RTI and 3.3% of GTI incidences. Resampling revealed mean and 90% confidence interval values of 10.9 (CI 6.9–14.5)% for RTI and 2.4 (0.6–4.4)% for GTI.

**Conclusion:**

The model created explained a relatively small proportion of the occurrence of RTI or GTI. Unpredictable exposure to disease agents, and individual susceptibility factors are likely to be key determinants of disease emergence. Yet, the model might be useful in prerandomization stratification of study population in RTI intervention trials where the expected difference between trial arms is relatively small.

**Trial registration:**

Registered at ClinicalTrials.gov with Identifier NCT00821509 on 12 March 2009.

**Electronic supplementary material:**

The online version of this article (doi:10.1186/s13063-016-1668-7) contains supplementary material, which is available to authorized users.

## Background

It is common knowledge that individuals in any population vary in their likelihood of contracting acute infectious disease. Inherited patterns of innate immunity responses [1, 2], variable adaptive immunity due to previous exposures to causative agents of the infections [3], and modification of the above responses by chronic diseases or their immune suppressive treatment [4] are each plausible explanations for a part of the observed differences in individual susceptibility. However, apart from a possible chronic disease record, this background information is not usually available for identifying suitable persons to participate in an infectious disease trial, or for trial-arm matching. In order to improve the harmonization, one can try to take advantage of expected post-randomization events affecting the emergence of infections including predicted frequency and intensity of exposures to infectious agents. Factors such as being a parent of young children regularly visiting a day care center, and occupational exposure to persons suffering from active infectious disease are often considered as risk factors for infectious disease in adults [5]. Yet, no generalizable data are available for relative roles or quantitative assessment of the different designated risk factors.

In this paper we describe an attempt to develop a prognostic model for predicting individual incidences of acute respiratory tract infection (RTI) or gastrointestinal tract infection (GTI) in the general adult population. The model is based on data collected in a controlled, cluster-randomized intervention trial evaluating the efficacy of intensified hand hygiene on occurrence of RTI or GTI in adult office workers in 2009–2010 in the Helsinki region, Finland (The STOPFLU Study). Occurrence of symptoms of RTI or GTI and that of recognized exposures to other persons with symptoms of RTI or GTI, were collected by weekly electronic reports. The protocol and the main results of the trial have been published before [6, 7], and selected parts are described in Additional file 1. In order to be able to homogenize the study arms in the original trial, interview data for various designated risk factors for acute infection were collected from the volunteer participants. In the current study, this questionnaire data was subjected to multivariate analysis in order to identify potential predicting factors, and significantly contributing variables were used in creating a tentative prognostic model. Model-predicted individual incidences of both infections and exposures were then calculated and compared with the observed ones.

## Methods

### Setup and general design of the background trial

In January 2009, altogether 1270 eligible office workers in 21 designated study clusters were offered a possibility to volunteer in a hand-hygiene intervention trial assessing the efficacy of two different hand-cleansing methods combined with transmission-limiting behavior in preventing acute infections (for details, see Additional file 1). No formal sample size determination was made. The aim was to enroll as many participants as possible. For reasons described in Additional file 1, additional participants were recruited throughout the 16 months duration of the trial. The study was not blinded to any counterpart.

### Data extraction and processing for the current study

#### Potential predicting variables

**Fig. 1:**
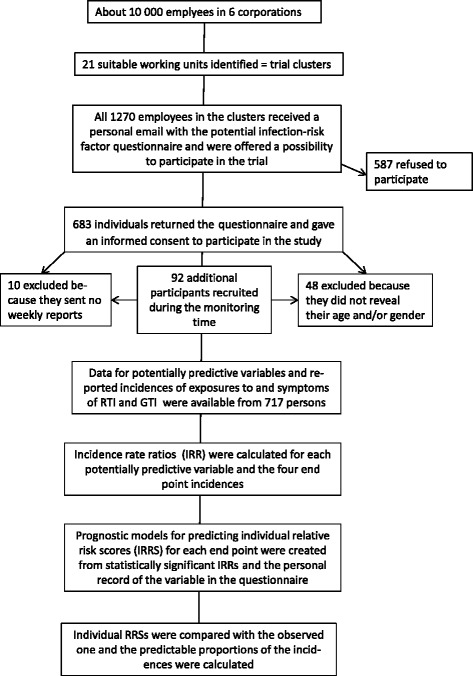
Flow chart of the current study

A flow chart describing the current study is shown in Fig. 1. As described previously by Savolainen-Kopra et al. [6] the information on designated infection-risk factors among the volunteer participants was collected through a standardized questionnaire. Its use for trial-arm harmonization in the original trial is described in Additional file 1. In the current analysis, all the data in the questionnaires, including age and gender, were used for potential predicting variable identification. Most of the questionnaire variables (Table 1) were treated as binary data and the participants accordingly divided in “yes” or “no” answering subgroups, separately for each question. Age (in years) was used as a continuous variable.

**Table 1:** Questionnaire for potential risk factors of acute infection^a^

Question/statement	Coding	Note
Age	Years	Continuous variable
Gender	Female = 1 Male = 2	
Trial arms	Control = 0 Soap = 1 Alcohol = 2	No specific hygienic instructions Hand washing with soap and water Hand cleaning with alcohol rub
No young children in the household	Yes = 0; No = 1	The question was negative
School-aged child(ren) in the household	Yes = 1; No = 0	
Younger than school-aged child(ren)	Yes = 1; No = 0	
Child(ren) in outside-home day care	Yes = 1; No = 0	
At-job-exposed adult in the household	Yes = 1; No = 0	A nonparticipant exposed in her/his work to sick children/patients with acute infections
Regular use of public transport	Yes = 1; No = 0	To and from the workplace
Chronic cardiovascular or respiratory disease	Yes = 1; No = 0	Requirement: physician-diagnosed
Influenza vaccination in autumn 2008	Yes = 1; No = 0	“Do not know” = 0
Business travel	Monthly or more = 1; Less = 0	Domestic travel to other cities or communities included
Smoking	Yes = 1; No = 0	Yes = currently smoking; No = never smoked or not anymore
Passive smoking	Yes = 1; No = 0	Exposure to cigarette/tobacco smoke due to other smokers

^a^Slightly modified from the original (Savolainen-Kopra et al. [6]) and simplified by omitting some details (Additional file 1)

#### Data for endpoint assessment

Throughout the intervention phase in the STOPFLU Study, weekly report forms (Additional file 1) were sent by email every Monday to the participants and they were requested to report, for the seven preceding days, possible daily symptoms of RTI or GTI, as well as whether they had experienced an exposure to other people obviously suffering from RTI or GTI. Typical symptoms of these infections were thoroughly described in the in-advance training phase and also repeated in every report request (Additional file 1).

The follow-up weekly report data on daily symptoms was first converted to weekly occurrence of symptoms. Thus, a calendar week with reported RTI symptoms in one or more days was defined as a week with RTI symptoms. A week with GTI was defined similarly. The reported exposure data were originally in the weekly format. The individual number of reported weeks with RTI symptoms was divided by the total number of reported follow-up weeks by the person in question and defined as the observed individual RTI incidence. The three other endpoint incidences, the observed individual incidences of GTI, and those of exposures to RTI or GTI, were defined similarly. According to the background trial protocol, intermittent stopping of reporting was ignored in these calculations. Likewise, data from individuals who stopped the reporting before the end of the trial (apparent dropouts) were included in the analysis.

### Statistical analysis

#### Identification of significant predicting factors

The mean incidences of reported RTI and GTI exposure and of symptoms were calculated for both “yes” and “no” answering subgroups of each question (Additional file 2).

A mixed-effect negative binomial regression model [8, 9], with backward variable selection using Akaike information criterion (AIC), was used to assess the impact of the in-advance chosen explanatory variables to the incidences of the reported RTI exposure, GTI exposure, RTI symptoms and GTI symptoms. The model was fitted to the individual level time-aggregated data taking into account the designated clusters and prerandomization matching in the data using random effects. The intervention arm was included in the model by adjusting its influence when estimating the effect of the baseline covariates age, gender, and the designated risk factors of the background trial [6] (Table 1). The negative binomial model allows estimating of the heterogeneity of individuals reflected in the autocorrelation and over-dispersion in the standard Poisson regression model [8, 9]. Interactions between age and gender were found to be negligible and, according to AIC [10], not necessary in the model.

Age was the only continuous variable. Its influence on the endpoints was tested in univariate analysis using the locally weighted regression method of R-statistics with the optimal LOESS bandwidth (0.5) chosen by generalized cross-validation.. We also made the analysis using fractional polynomials testing the effect of various exponential functions on the performance of the model but did not find significant difference between the models (AIC difference less than 1). For reasons described in the “Results” section, the participants were arbitrarily divided in three subgroups, 20–30 years, 31–40 years, and more than 40 years of age, respectively for a descriptive analysis.

#### Prediction models and their testing

We used the final models to suggest a variable-specific incidence rate ratio (IRR) for each endpoint. Relative predictive risk scores (RRS) were calculated for each individual and each of the four endpoints using a sum formula taking into account the variable-specific IRRs and the presence of the indicated variables in the designated risk factor record of the person in question. A general formula used is shown below:

$$ RRSa=b+{v_1}^a\kern0.28em \mathrm{x}\kern0.24em {v_2}^a\mathrm{x}\dots x\kern0.28em {v_i}^a $$

where *RRSa* is the relative risk score for participant “a,” *b* is the baseline, *v*_*i*_ is the mean IRR of a significant variable, and the exponential ^*a*^ refers to the presence of this variable in the questionnaire record. If present, the value given to the exponent was 1, otherwise 0. For *v* = age, the IRR was the increment per year and the exponent age in years – 20. The baseline was in practice excluded from the calculations because it is case-specific and time-depending and, therefore, not suitable for a general prediction model. Significant variables into the models were selected on the basis of AIC assessment [10]. Performance of the models was tested by comparing the predicted sum scores with the corresponding observed endpoint incidences. The proportion of the incidences explained by the variables in the model was assessed using the Cox-Snell multivariate formula (Pseudo R^2^) [11] – with and without normalization. To assess the effect of “zero inflation,” due to majority of participants reporting no person weeks with GTI symptoms, we repeated the analysis by using a zero-inflated negative binomial model by using the Vuong test [12].

For internal validation of the model we used bootstrapping with 100 iterations revealing mean values and confidence intervals for the fraction of endpoint incidences explained by the models. Possible overfitting of the models was tested by calibration according to Pavlou et al. [13].

## Results

### General observations

Out of the 1270 eligible employees of the six participating corporations altogether 683 volunteers of the background trial filled the prerandomization questionnaire on personal features and living conditions assumed to influence the risk of capturing RTI or GTI during the trial. None of the volunteers was excluded from the trial. During the trial, 92 additional volunteers were recruited into the study. Out of the 775 registered participants, 10 persons did not send any weekly reports of exposures or own symptoms, and 48 persons were excluded from the current analysis as they did not reveal their gender. Thus, there were altogether 717 persons with both questionnaire data, including the age and gender, and follow-up reports on possible infections and exposures (Fig. 1). Personal answers by the participants to the questionnaire were recorded as such and not checked from possible original documents, and the data on exposures and symptoms were based on self-reporting only. As reported before [7], out of the 38,644 follow-up weekly reports evaluable, 10,817 included a reported exposure to RTI, and 2510 to GTI, while symptoms of RTI were reported to have occurred during 4662 weeks and those of GTI during 869 weeks. The mean age (43.7 years, SD 10.5, range 20–64) and the female to male ratio (about 3:1) of the participants were also reported before [7].

### Age and gender predicted both reported exposure and disease symptoms

**Fig. 2:**
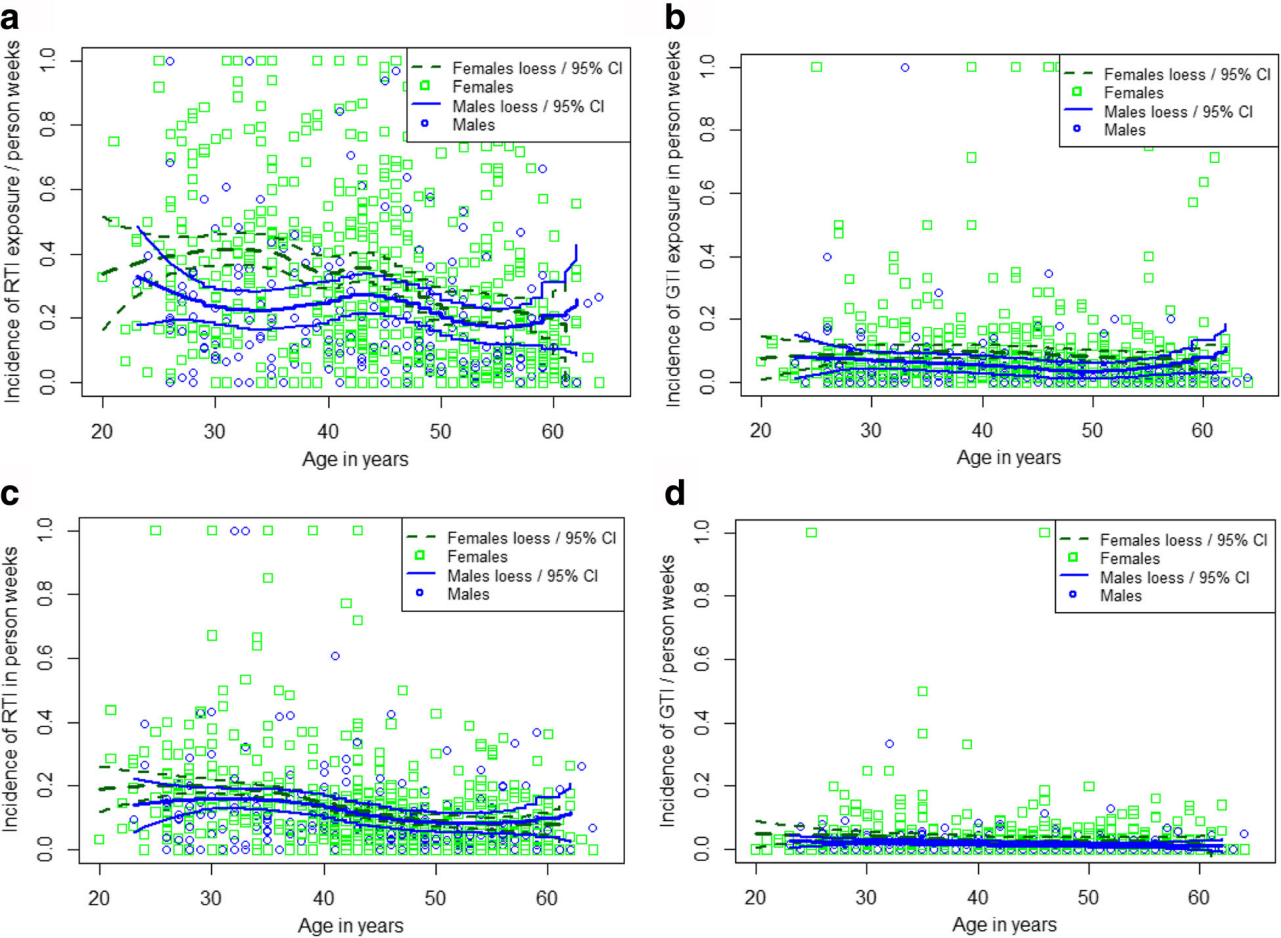
Age-dependence of the analyzed endpoints. Incidences of reported exposures to persons with obvious respiratory (RTI, **a**) and gastrointestinal tract infections (GTI, **b**), and those of weeks with reported own RTI (**c**) or GTI symptoms (**d**). Squares and circles stand for individual values of women, and men, respectively. The locally weighed regression (LOESS) functions with 95% confidence intervals were calculated with the R-statistics separately for the two genders

The raw data on distribution of weeks with reported exposures to GTI or RTI, and of weeks with reported symptoms of GTI or RTI, among subgroups generated according to the answers to the questionnaire are tabulated in Additional file 2. Both age and gender had a significant effect on the endpoint incidences. Increasing age appeared to protect from both exposures and symptomatic disease while female gender seemed to increase both the incidence of exposures and that of GTI disease (Fig. 2). Other variables with significant effect on endpoint incidences according to a univariate analysis are shown in Table 2. Young children in the household, especially if in outside-home day care, not surprisingly increased both reported exposures to, and symptoms of, both RTI and GTI. This increase was, however, not confirmed in the mixed-effect multivariate analysis (Table 3). Out of the four endpoints, the strongest effect by children in day care was seen on the incidence of weeks with RTI symptoms (IRR 1.328; CI 0.966, 1.432; *p* = 0.07).

**Table 2:** Variables, excluding age and gender, significantly associated with relatively increased incidence of reported exposures or reported weeks with symptoms of respiratory tract (RTI) or gastrointestinal infections (GTI) in univariate analysis

Variable	Incidence rate ratio, confidence interval (CI) of endpoint, and significance			
	Weeks with reported exposure to		Weeks with reported symptoms of	
	RTI	GTI	RTI	GTI
Young children in the household	1.174 CI 1.031, 1.338 *p* = 0.02	1.228 CI 1.036, 1.457 *p* = 0.02	1.093 CI 0.951, 1.257 *p* = 0.21	1.023 CI 0.812, 1.290 *p* = 0.85
Children not yet in school	1.244 CI 1.029, 1.505 *p* = 0.02	1.335 CI 1.048, 1.702 *p* = 0.02	1.288 CI 1.054, 1.575 *p* = 0.01	1.323 CI 0.958, 1.323 *p* = 0.09
Children in outside-home day care	1.239 CI 1.023, 1.501 *p* = 0.03	1.409 CI 1.099, 1.806 *p* = 0.01	1.384 CI 1.132, 1.692 *p* = 0.00	1.383 CI 0.997, 1.918 *p* = 0.05
Living with at-job-exposed adult	0.909 CI 0.699, 1.181 *p* = 0.48	1.328 CI 0.947, 1.861 *p* = 0.10	0.867 CI 658, 1.143 *p* = 0.31	0.948 CI 603, 1.490 *p* = 0.82
Regular use of public transport	1.071 CI 930, 1,235 *p* = 0.34	1.061 CI 0.869, 1.296 *p* = 0.56	1.163 CI 1.002, 1.349 *p* = 0.051.163	1.236 CI 0.976, 1.565 *p* = 0.08
Suffering from chronic respiratory or cardiovascular disease	1.080 CI 880, 1,327 *p* = 0.46	1.044 CI 0.799, 1.364 *p* = 0.75	1.285 CI 1.039, 1.590 *p* = 0.02	1.271 CI 0.902, 1.791 *p* = 0. 17
Recipient of seasonal influenza vaccine in autumn 2008	1.071 CI 0.894, 1.282 *p* = 0.46	1.052 CI 0.831, 1.332 *p* = 0.67	1.312 CI 1.088, 1.582 *p* = 0.00	1.108 CI 0.814, 1.508 *p* = 0.52
Passive smoking	1.141 CI 1.000, 1.302 *p* = 0.05	1.222 CI 1.029, 1.453 *p* = 0.02	1.009 CI 0.876, 1.162 *p* = 0.90	1.144 CI 0.910, 1.437 *p* = 0.25

**Table 3:** Variables significantly predicting relatively frequent reported exposure to or reported own symptoms of respiratory (RTI) or gastrointestinal tract infection (GTI)

Variable^a^	Incidence rate ratio, confidence interval (CI) and *p* value			
	Reported exposure		Reported own symptoms	
	RTI	GTI	RTI	GTI
Increasing age	Component I 1.064 CI 1.013,1.118 *p* = 0.014 Component II 0.999 CI 0.998, 1.000 *p* = 0.001	0.984 CI 0.975, 0.992 *p* < 0.001	0.975 CI 0.968, 0.981 *p* < 0.001	0.983 CI 0.972, 0.994 *p* = 0.003
Male gender	0.701 CI 0.597, 0.823 *p* < 0.001	0.614 CI 0.494, 0.763 *p* < 0.001		0.695 CI 0.530, 0.911 *p* = 0.009
Child in day care			1.176 CI 0.966, 1.432 *p* = 0.107	
Use of public transport			1.204 CI 1.043, 1.389 *p* = 0.011	
Chronic disease			1.215 CI 0.990, 1.491 *p* = 0.062	1.284 CI 0.915, 1.804 *p* = 0.15
Recipient of influenza vaccination			1.328 CI 1.112, 1.586 *p* = 0.002	
Living with at-job-exposed adult	1.111 CI 0.853, 1.446 *p* = 0.43	1.752 CI 1.240, 2.475 *p* = 0.001		
Passive smoking	1.173 CI 1.033, 1.332 *p* = 0.014	1.261 CI 1.065, 1.494 *p* = 0.01		

^a^Empty spaces indicate that the variable was not significant in predicting the corresponding endpoint. The following variables did not contribute significantly to any endpoint: living with young children of any age, or with a child in the school age; frequency of business travel; active smoking

**Fig. 3:**
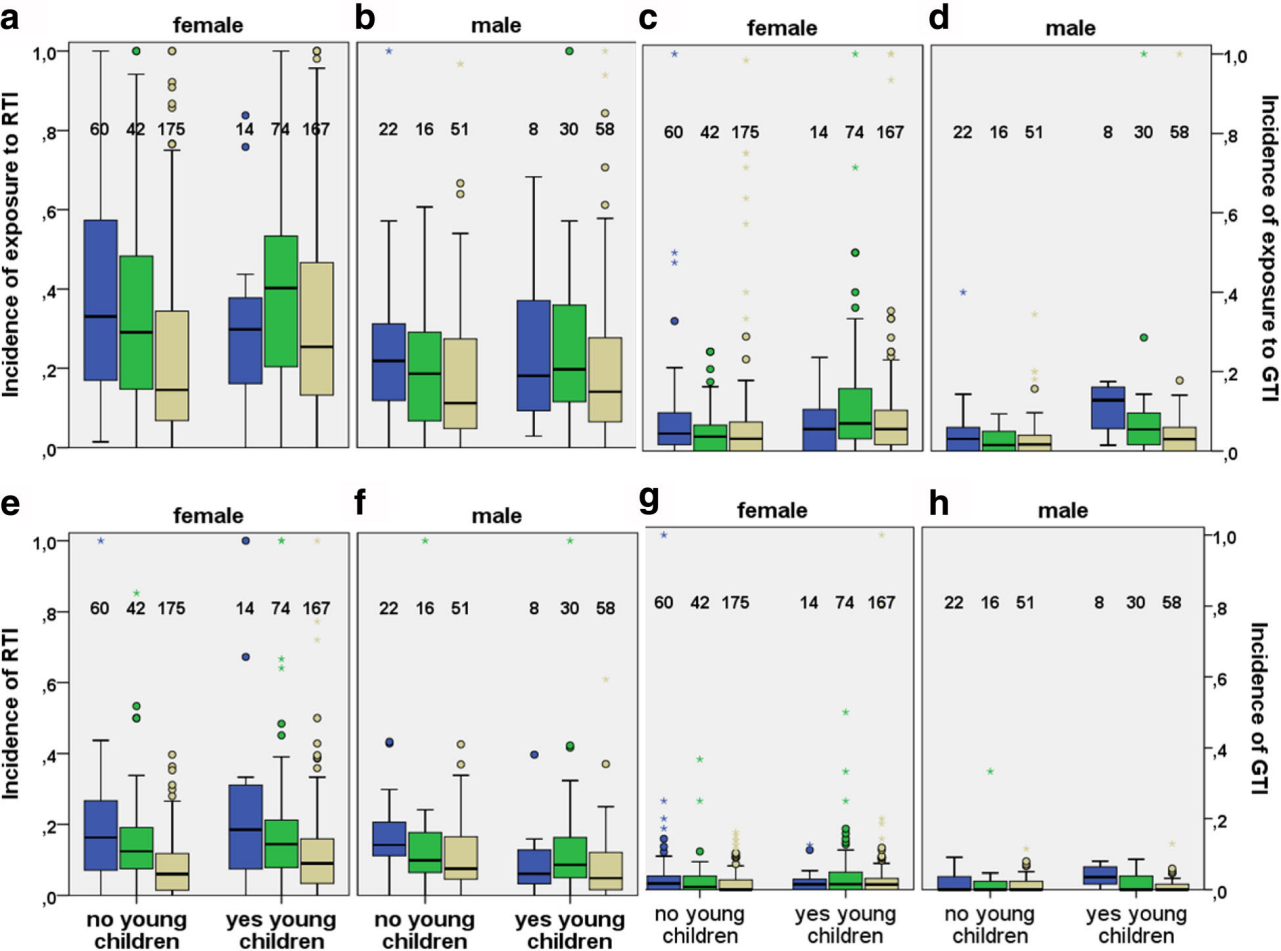
Apparent effects of gender, age, and children in the household on reported exposures and infections. The population was divided arbitrarily into three age groups shown by colors: blue, below 30 years; green 31–40 years; ochre, over 40 years. The boxes (limits 25 and 75%) include a horizontal median line if differing from the bottom line. In addition, individual outlier values and numbers of participant in each subgroup are shown. Panels **a** and **b**, reported exposures to people with RTI symptoms; **c** and **d**, exposures to GTI; **e** and **f**, weeks with reported own RTI symptoms; **g** and **h**, reported GTI symptoms

Interestingly, the age-dependence of reported exposures to RTI showed two components, first a moderate increase, and thereafter a slow but significant decrease (Table 3). The breaking point of the two components was calculated, by estimating the point of zero derivative of the estimated age profile function, to be at about 32 years. Because of this observation and with respect to the common presence of young children in the households of the younger adult age groups the participants were divided arbitrarily into three age groups, 20–30 years, 31–40 years, and 41 years or older. The incidences of reported exposures, and those of reported RTI or GTI, were then compared between the age groups, separately for women and men, and according to the presence or absence of young children in the household. As seen in Fig. 3, the age-dependence of both reported exposures and reported weeks with symptoms is apparent, in both genders, whether there were young children in the household or not.

Out of the other tested variables, the multivariate analysis confirmed a significant effect for RTI (but not GTI) disease for two variables, [1] regular use of public transport to and from the workplace, and [2] history of seasonal influenza vaccination. In addition, suffering from physician-diagnosed cardiovascular or respiratory disease increased the IRR almost significantly (IRR 1.215; CI 0.990, 1.491; *p* = 0.062) (Table 3). Two variables, living with a nonparticipant adult who presumably had a high exposure risk to RTI or GTI in their job, and passive exposure to tobacco smoke, were associated with relatively increased reported exposure incidence to GTI and the latter also to RTI, but had no effect on the reported incidences of either disease in the study participants.

As the data were derived from a three-arm intervention trial, the analysis was carried out both including the information on trial arm and without the arm variable in the model. The contribution of the other variables to the endpoints was found to be practically identical in both cases.

### Explanatory power of developed prediction models

The prediction models for calculation of the individual RRSs of the different endpoint incidences were as follows:

A. RRS for RTI exposure = 0.701^Male^ × 1.111^Living with at-job-exposed adult^ × 1.173^Passive smoking^ × 1.064^(Age−20)^ × 0.999^(Age−20) × (Age−20)^;
B. RRS for GTI exposure = 0.614^Male^ × 1.752^Living with at-job-exposed adult^ × 1.261^Passive smoking^ × 0.984^(Age−20)^;
C. RRS for RTI = 1.176^Day care^ × 1.204^Use of public transport^ × 1.215^Chronic disease^ × 1.328^Recipient of influenza vaccination^ × 0.975^(Age−20^);
D. RRS for GTI = 0.695^Male^ × 1.284^Chronic disease^ × 1.328^Recipient of influenza vaccination^ × 0.983^(Age−20)^.

The variables included in the models were those found to be associated with statistically significant IRRs in the multivariate analysis (Table 3) as well as other variables found to increase the AIC value if left out from the model [10]. In addition, the children in outside-home day care subgroup was included in the score calculation of RTI infections as it showed a significant effect in the univariate analysis (Table 2).

**Fig. 4:**
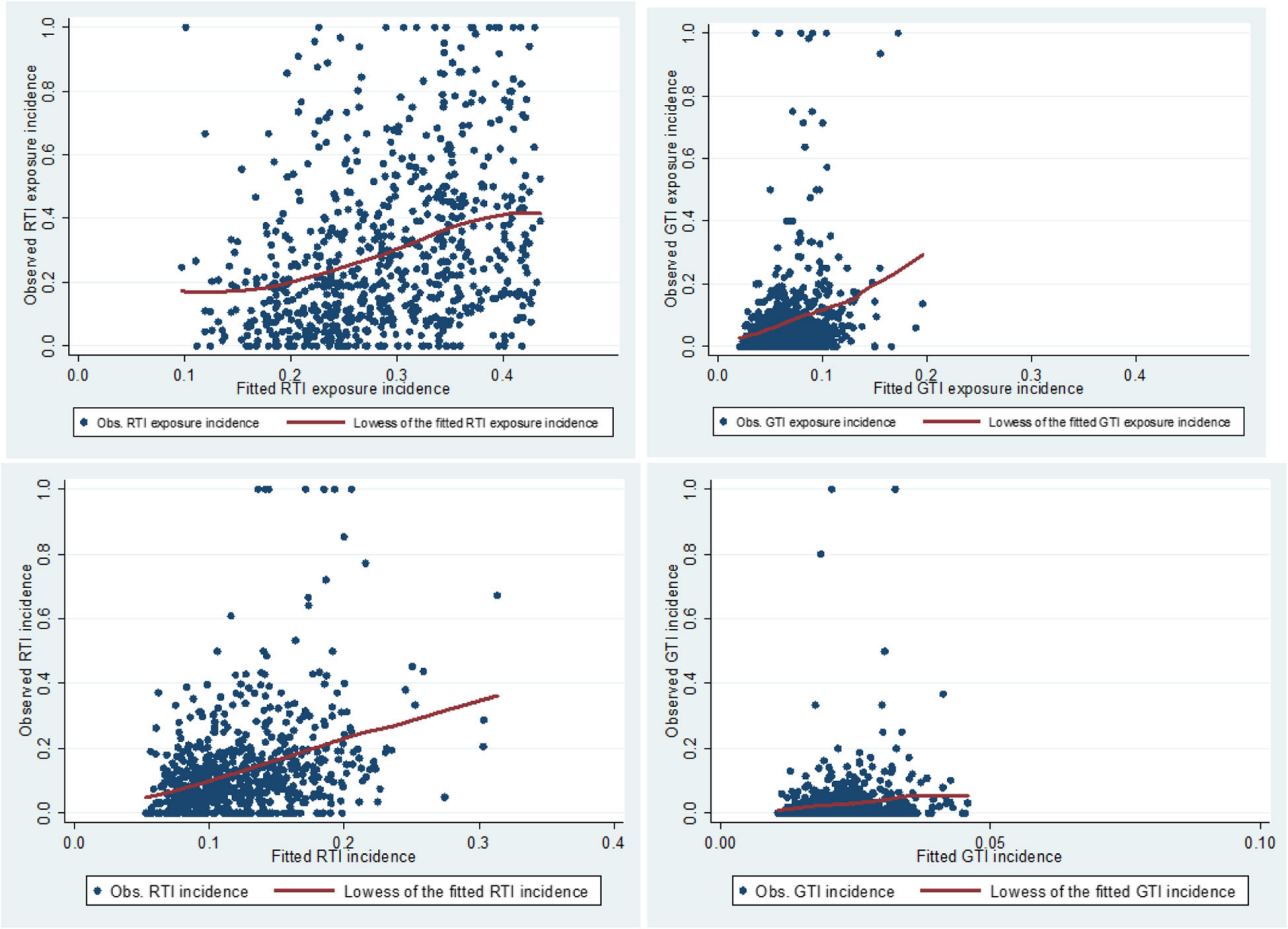
Correlation of model-predicted relative risk scores with observed incidences of different endpoints. Exposures to respiratory tract infection (RTI) (*left upper* panel), of reported weeks with RTI symptoms (*left lower*), of exposures to gastrointestinal tract infection (GTI) (*upper right*), and of reported weeks with GTI symptoms (*lower right*). Locally weighted regression is shown by LOESS curves

Plotting of the obtained individual RRSs against the corresponding observed incidences showed a roughly linear relationship but with a wide range of variation (Fig. 4). According to the Cox-Snell formula [11] the fit of the model to the data was moderate or poor: the model explained 11.2% and 3.3% of the RTI or GTI symptom weeks, respectively. The analysis was repeated with a normalized version of the Cox-Snell formula, but the obtained values were very close to those from the un-normalized version. Resampling of the data for 100 times showed a mean value of 10.9 (CI 6.9–14.5)% for RTI and 2.4 (CI 0.6–4.4)% for GTI. So the optimism in the original sample is not very large. In a test for possible overfitting of the model the calibration slope for RTI was fairly good, 0.90 (CI 0.86, 0.94), but for GTI only 0.61 (CI 0.58, 0.64) suggesting significant overfitting.

For weeks with reported exposure to RTI or GTI, the corresponding explaining-power figures were 9.3% and 5.4%, respectively. These were not confirmed by resampling. To assess the effect of possible “zero inflation,” due to the majority of participants reporting no person weeks with GTI symptoms, we repeated the analysis by using a zero-inflated negative binomial model, but this did not improve the fit (by using the Vuong test [12]). In another analysis the reported incidences of exposures to RTI or GTI were compared with reported incidences of homologous infections. The exposures explained 13.9% and 5.9% of reported weeks with RTI or GTI symptoms, respectively.

## Discussion

In this paper we describe an attempt to develop prognostic models for predicting future incidences of acute respiratory or gastrointestinal tract infections in the general adult population. Both the potential predicting factors and the endpoint data used were derived from a previously published [7] hand-hygiene intervention trial carried out in Finland in 2009–2010. The potential predicting variables tested here were based on a prerandomization questionnaire used in the trial in order to harmonize the trial arms. Age, gender, chronic cardiovascular or respiratory disease, regular use of public transport, and history of seasonal influenza vaccination significantly influenced the subsequent GTI and/or RTI incidences. However, prognostic models based on these factors had only a moderate predicting power as regards the RTI incidence and a poor predicting power in the case of GTI incidence.

Caveats relating to data based on self-reporting only, and not confirmed in individual cases by observations or examinations by trained health care personnel, have been discussed before [7]. A questionnaire enquiring into a range of personal matters in the context of an occupational health trial may also have been felt too intimate and, in some persons, result in intentional or unintentional adjustment of the answers. The emergence of a symptomatic acute infection in the respiratory or gastrointestinal tract requires a transmission of an infectious agent, usually from another person, but a relevant exposure can be unrecognizable, due to an asymptomatic infection in the contact person [14], or can for some reason remain unnoticed, ignored, or forgotten by the time of the weekly reporting.

Another weakness of the current study is its post hoc nature which results, among other things, in the fact that some of the generated variable-specific subgroups are likely to be too small to provide sufficient power to detect all possibly relevant influences. A further point to note is a possible selection bias. Only about half of the eligible employees in the trial clusters volunteered to participate in the study. Reasons for declining the invitation to the study were not examined but one can assume that there may be differences in the health behavior and other personal properties between these “silent” and the active, reporting members of the trial clusters. As a consequence, we cannot be sure how well the results of the current study represent the general adult population.

A common practice in analyzing clinical trials is to present the results in both “per-protocol” and “intention-to-treat” modes with the latter often also including both dropouts and cases with missing supplementary data. The STOPFLU Study protocol included a plan to include in the analysis those with intermittently lacking weekly reports (usually due to holidays, but in practice, for any reason), participants recruited after the onset of the intervention (“missing” early data), as well as reports from participants who ceased to report before the end of the trial (“dropouts”). The latter was expected to occur relatively frequently because the employing corporations of the participants were in the middle of reorganization (Additional file 1). In spite of their per-protocol nature these defects in the data require comments for potential influence on the developed models. Missing data in the potential predicting variable questionnaire were minimal. Usually only one or a few persons out of 717 had declined answering a given question. An exception was “passive smoking” which was not answered by nine persons (Additional file 2). It is highly unlikely that these omissions could influence the outcome, and thus they were ignored in the model building. Intermittent breaks in weekly reporting were relatively common but assumed to take place at random and, therefore, also excluded from the model variables. Missing data, due to delayed enrollment or premature ceasing of reporting, concerned about 30% of the participants. The dates of the delayed enrollment were distributed over the duration of the trial (Additional file 3) and are, therefore, unlikely to influence the outcomes significantly. Ceasing of reporting was almost always related to stopping working for the previous employer or due to transfer to another working unit. Therefore, we initially considered that it was not endpoint dependent and thus not affecting the accuracy of the outcomes. According to a regression analysis [15], ceasing the weekly reporting was not dependent on reported endpoint incidences, and thus, from a statistical point of view, occurring at random. The entire duration of the trial was 16 months but for some individuals the total reporting time was relatively short (Additional file 3). Since in theory, short reporting periods might influence the endpoints if occurring at a low or very high epidemic season, we repeated the analysis for identifying significant predictor variables by excluding participants with fewer than 50 weekly reports. The resulting variable-specific IRRs did not much differ from those of all reported data although the *p* values in the case of GTI increased beyond the significance limit (see Additional file 3).

The overall predicting power of the models developed was at best only moderate for RTI and poor for GTI. This was not surprising after seeing the IRRs of the identified significant predicting variables which differed relatively little from 1 and had broad confidence intervals. The tested variables were no better in predicting the incidence of exposures to other persons with RTI and only vaguely for exposures to GTI. Temporally associated exposures were previously reported to have a strong relation to homologous disease in the reporter [14] but the temporal association cannot be predicted in studies of long duration. Out of the different factors included in the analysis, only a few showed a statistically significant contribution to the endpoint. Women had reported relatively more exposures both to GTI and RTI as well as weeks with GTI symptoms than men. We believe that this difference was most likely based on plausibly different behavior of the genders rather than caused by a true gender-related biological factor. It is possible that women have had more contacts with other people during their free time than men. Women may also be relatively more sensitive to recognize and report possible exposures. As regards GTI, the observation might also reflect real life as it is possible that women, as mothers, are often in more close contact with their sick children, a likely source of GTI in many cases. Through most of the age ranges of the participants, increasing age appeared to reduce the risk of exposure to both GTI and RTI as well as that of the emergence of RTI and GTI symptoms. The observed influence of age on the incidences of both exposure and infection is, as such, understandable. Younger people move around more than the older ones during their free time and have more contacts with other people. It is also possible that younger people are relatively more sensitive to recognize and report possible exposures and to notify symptoms of infection. Of course, accumulating acquired immunity to some of the infectious agents could contribute to this decreasing trend of symptomatic RTI and GTI.

Three other variables were associated with an increased RTI incidence. Various surfaces in the public transport vehicles may serve as invisibly contaminated fomites, and infections may be transmitted manually without recognition of the exposure [16, 17]. As short distances to infected other persons in the vehicles are difficult to avoid and, as respiratory viruses may also be spread via aerosols without coughing [16–18], it is understandable that using the transport was found to increase the risk of RTI. Chronic cardiovascular or respiratory disease is a known risk factor for complications of influenza and most likely for other respiratory viral infections [19]. Hence, the almost significantly increased incidence of weeks with reported RTI symptoms but without an increase in the reported exposures is not unexpected. Influenza vaccine recipients during the preceding season partially overlapped the above subgroup suffering from chronic disease (these people get the influenza vaccine cost-free in Finland). Possible specific reasons for taking the vaccine were not enquired, but influenza vaccine is recommended by the health authorities to people who want to get a protection against epidemic influenza. It is possible that the remaining vaccine recipients have historically suffered from frequent colds and have, therefore, obtained the vaccine. Anyhow, as above, a slightly increased incidence of RTI is not surprising. A protection effect through the trial was not expected, as virologically documented influenza was a minor element among the tested, relatively severe RTI during the winter 2009 epidemic [7], and a vaccine received in autumn 2008 had no effect against the H1N1 virus pandemic during the following influenza season.

We were somewhat surprised to see that the statistical multivariate analysis revealed a lack of influence for two designated cluster-risk sum calculations: young children in the household and smoking. It is common knowledge that children suffer from acute RTI and GTI more frequently than adults and might easily transmit the infection to their parents who are often living in close physical contact with sick children. In the univariate analysis, having under-school-age children in the household or a child in outside-home day care were both associated with IRRs significantly above 1. The stronger effect by the age and gender may have diluted out these effects in the multivariate analysis. It is possible that a larger proportion of households with children in these groups could also have rendered the difference significant in the multivariate model. One hundred and eighty-five participants (about one quarter) lived in a household with young children, and in 95 out of 716 households (13.3%) at least one of the children was under school age.

Smoking was another variable where we expected, but did not obtain, an effect on the incidence of respiratory symptoms. It is well-established that smokers suffer from both acute and chronic respiratory symptoms more than nonsmokers of the same age [20, 21]. The reasons for our failure to see the expected effects can only be speculated. According to the questionnaire answers, 150 out of 714 participants (21%) were smokers, a number well-corresponding to the known proportion of smokers in the general population in Finland [22]. However, only 18 of the participants of the study can be considered as heavy smokers (more than 20 cigarettes per day).

Living with an adult nonparticipant, who might carry home infections from their job, and passive smoking were associated with increased reported exposure incidences but not with those of RTI or GTI symptom weeks. One can speculate that a recognized exposure has resulted in intensified hand hygiene and that the person has succeeded in avoiding the consequent symptomatic infection. Another possible explanation is a situation-prompted increased sensitivity to recognize and/or register the exposures. On the other hand, passive smoking is an irritating situation to most people who are not smoking themselves, and may result in a sort of over-sensitivity to detecting symptoms of disease in smokers.

## Conclusion

We designed a tentative model for calculating the risk of acute infection, based on a combined effect of age, gender, and a few other fixed variables which were found to significantly influence the incidence of acute infections in adult participants of a hand-hygiene intervention trial. The incidences predicted by the model were compared with those reported during a 16-month monitoring period. The model explained 11.2% of the reported RTI incidence but only 3.3% of that of GTI. This suggests that a majority of the emerging disease events are based on a combination of temporally associated exposure to disease agents, chance and unknown individual susceptibility/resistance factors. While it seems that for trials involving GTI these models are not likely to be useful, it might be worthwhile to consider using the fixed variables found to be significant in this study for prerandomization stratification of participants recruited into trials on RTI with relatively small expected difference between trial arms.

## Additional files

Additional file 1: Selected features of the data source, a controlled, cluster-randomized intervention trial on the efficacy of intensified hand hygiene in preventing acute infection (the STOPFLU Study). A brief description of general setup of the trial, questionnaire for designated risk factors, interventions, weekly report forms, and key definitions. (PDF 300 kb)
Additional file 2: Raw data on the relationships between designated risk factors and occurrence of infections. Distribution of weeks with reported exposures to GTI or RTI, and of weeks with reported symptoms of gastrointestinal infection (GTI) or respiratory tract infection (RTI), among subgroups generated according to the answers to the questionnaire. (XLSX 32 kb)
Additional file 3: Additional descriptive and statistical analyses. Time distribution of enrollment of study participants, frequency of various monitoring periods, and reanalysis of potential significant predicting variables using a dataset with participants reporting data for 50 weeks or more. (PDF 237 kb)

## Abbreviations

AIC: Akaike information criterion
GTI: Gastrointestinal tract infection
IRR: Incidence rate ratio
RRS: Relative risk score
RTI: Respiratory tract infection
