# Supplementary material for: Development of a prognostic model based on demographic, environmental and lifestyle information for predicting incidences of symptomatic respiratory or gastrointestinal infection in adult office workers
Source: Trials. 2016 Nov 16;17:545. doi: 10.1186/s13063-016-1668-7 (PMC5112653; doi:10.1186/s13063-016-1668-7)
Supplement: Additional file 1: — Selected features of the data source, a controlled, cluster-randomized intervention trial on the efficacy of intensified hand hygiene in preventing acute infection (the STOPFLU Study). A brief description of general setup of the trial, questionnaire for designated risk factors, interventions, weekly report forms, and key definitions. (PDF 300 kb) [file 13063_2016_1668_MOESM1_ESM.pdf]

Additional file 1.

Selected features of the data source, a controlled, cluster randomized intervention trial on the efficacy of intensified hand hygiene in preventing acute infection (STOPFLU Study).

(Contains direct extracts or slightly modified copies from items in references 1 or 2

1. ***Purpose and setup of the trial***

The aim of the study was to investigate whether standardized instructions for transmission reducing behavior and intensified hand cleansing could reduce the occurrence of acute respiratory tract (RTI) or gastrointestinal the Helsinki region, Finland. Although all the corporations had an occupation health clinic with physicians and nurses serving the staff, data for this study was collected by self-reporting in order to reduce the disturbance of the daily work of the participants. Twenty-one study clusters each consisting of distinct groups of office workers with potential daily contacts within the group but relatively little with colleagues outside the group were identified in the corporations. The duration of the trial was 16 months, from February 2009 to May 2010, in order to cover as many as possible of the different causative agents with variable seasonal intensity of the transmission.

The general features and the main results of the trial have been described before (1,2).The study protocol (1) was accepted by the Institutional Review Board of the National Public Health Institute, Finland (KTL) (9/2008) and registered at ClinicalTrials.gov (http://clinicaltrials.gov/) with an identifier of NCT00821509, 12 March 2009. Twenty-one clusters of potential participants were identified among office workers in six corporations in the Helsinki Region. Chief physicians of the occupational health clinics serving the staff in each of these corporations evaluated and approved the protocol, after consulting the Health and Safety Committees of the following corporations: Kesko Oyj, Outokumpu Oyj, Outotec Oyj, Nordea Bank Finland Plc, SOK group, and S-Bank. Only employees who gave an informed consent were enrolled into the study. The aim of the study was to investigate whether standardized instructions for transmission reducing behavior (such as coughing and sneezing on sleeve rather than on hands, and avoiding hand shaking) and intensified hand cleansing could reduce the occurrence of RTI or GTI in adult office workers. The two intervention arms differed in the method of hand cleansing. The participants in one arm were recommended to use frequent washing with soap and water, in the other rubbing with ethanol-based disinfectant was used instead. The third arm served as a control with no subsequent instructions related to hand hygiene. At the later stages of the planning, the international economic recession also hit some of the participating enterprises resulting in insecurity in the expectations of keeping the permanent staff. Therefore, the number of dedicated working groups in the enterprises remained much lower than what was initially anticipated. For the same reason, it was anticipated that through the duration of the trial, there will be turnover in the staffs affecting our study clusters. Therefore we decided to analyze the data as incidences and accept data from interrupted follow-ups and that from new "replacement" participants recruited during the study. No formal sample size determination was done. We simply tried to enroll as many participants as possible. The study was not blinded to any counterpart.

2. *Trial arm harmonization*

It is common knowledge that individuals vary in the occurrence of acute RTI and GTI, and in order to minimize the potential confounding effect of this phenomenon in the intervention trial we tried to harmonize the trial arms by stratification before cluster randomization. In the absence of applicable guidance from the literature the factors included in the formula were selected by the research group through "common sense".

The eligible participants, 1270 office workers, received a questionnaire (see below) enquiring living conditions and personal features with potential effect on expected exposure to infectious agents or of susceptibility to RTI or GTI (Additional file 1 in ref 1; retyped for space saving).

---

1. Working unit

A drag down menu with all participating units

2. Sex

( ) male ( ) female

3. Age (years) ____

POSSIBILITIES OF CONTAGION OUTSIDE WORK

4. Residents in the same household

4.1 [ ] no school aged or younger children
4.2 [ ] one or more school aged children
4.3 [ ] one or more children under school age
4.4 [ ] at least one child under school age attending day care/ preschool/ weekly club etc.
4.5 [ ] grown-up exposed to children with cold or vomiting/diarrhea disease at work in school, kindergarten, children's club, health care etc.

5. I use mainly public transportation for commuting to work ( ) yes ( ) no

6. I have a medically diagnosed chronic heart condition, atherosclerosis, asthma or other respiratory disease

( ) no ( ) yes, what? __________________

7. I received influenza vaccination in autumn 2008

( ) yes ( ) no ( ) I do not know

8. My work normally includes trips to other municipalities

( ) weekly ( ) monthly ( ) more rarely or not at all

9. Smoking

9.1 ( ) I have never smoked
9.2 ( ) I do not smoke any more
9.3 I smoke in average
( ) <10 cigarettes/cigars/pipes a day
( ) 10-20 cigarettes/cigars/pipes a day
( ) >20 cigarettes/cigars/pipes a day

10. If you answered "I do not smoke any more" to the previous question, answer to the following

I started smoking year________

I quit smoking year __________

11. Exposure to cigarette smoke of other smokers

( ) not at all
( ) infrequently
( ) daily, how many hours a day in average ____

Individual answers to the above questions were converted to designated weighted cluster sums for the 21 study clusters as follows: First, the occurrence of each designated risk factor in a given cluster was related to the frequency of the factor in the entire population interviewed. Next, these ratios were inter-connected to reveal an arbitrary transmission risk sum score for the cluster using the following formula:

Score for each unit = round (10000 * (s1/m1+ 2*s2/m2 + s3/m3 + s4/m4 + s5/m5 + s6/m6) / n)

where s1-s6 denote corresponding unit-wise sums of each indicator, m1-m6 corresponding total sums over all clusters, i.e. border sums describing frequency of a given variable in the complete material and n, number of people in the cluster.

Indicator variables included:

x1 school aged or younger children (questions (Q) 4.2 or 4.3 in Table 1)
x2 at least one child under school age attending day care/ pre-school classes/ weekly club (Q4.4)
x3 smoking (0 = if 1 or 2, otherwise > 1(Q9.3)
x4 medically diagnosed chronic heart condition, atherosclerosis, asthma or other respiratory disease (Q6)
x5 mainly use of public transportation for commuting to work (Q5)
x6 grown-up exposed to children with cold or vomiting/diarrhoea disease at work in school, kindergarten, children's club, health care etc (Q4.5)

The clusters were subsequently stratified according to the rank of the risk sums and randomized into the three study arms in blocks of three (1). During the 16 months of follow-up, the participants were weekly requested to report occurrence of respiratory or gastrointestinal symptoms and observed exposures to other persons obviously suffering from RTI or GTI.

As reported before (2) the calculated cluster risk sum did not correlate with the incidence of reported infections in the cluster in any STOPFLU trial arm suggesting that the designated risk factors included, or the weighting of different factors used, were not correct in the cluster level analysis.

3. *Reporting of exposures, own symptoms, and days off on electronic forms*

The standardized reporting form (below) was sent by email to all participants on Monday morning (1). If an answer with filled form was not received by Friday on the same week, a reminder was sent to the participant in question. The reminder was not sent if the participant had informed temporary stop in reporting because of holiday or other reason.

Reported week (the past week) Automatically given **Working unit** Automatically given

Exposure during the past week (I was around people who had symptoms of respiratory infection or vomiting/diarrhea disease)

	Exposure				
	at work	during work trip	at home	elsewhere during free time	No exposure to my knowledge
respiratory infection	[ ]	[ ]	[ ]	[ ]	[ ]
vomiting/diarrhea disease	[ ]	[ ]	[ ]	[ ]	[ ]

Own health condition and possible absence from work during the past week

[ ] I was healthy and normally at work the whole week or on vacation
[ ] I had symptoms of respiratory infection during the past week
[ ] I had symptoms of vomiting/diarrhea disease during the past week
[ ] I was absent from work at least one day due to another reason

Choose the most appropriate alternative for each day

"Symptoms" mean symptoms of respiratory infection or vomiting/diarrhea disease

	Healthy at work or out of work as designed	At work with symptoms	Symptoms, but not supposed to be at work	Part of the day at work with symptoms	Absent due to symptoms	Absent due to child's symptoms; own symptoms as well	Absent due to child's symptoms; self healthy	Other reason for absence
Monday	( )	( )	( )	( )	( )	( )	( )	( )
Tuesday	( )	( )	( )	( )	( )	( )	( )	( )
Wednesday	( )	( )	( )	( )	( )	( )	( )	( )
Thursday	( )	( )	( )	( )	( )	( )	( )	( )
Friday	( )	( )	( )	( )	( )	( )	( )	( )
Saturday	( )	( )	( )	( )	( )	( )	( )	( )
Sunday	( )	( )	( )	( )	( )	( )	( )	( )

4. Definitions

(Translated from original Finnish versions)

Respiratory infection

A person will be defined to have an acute respiratory tract infection if she/he is having at least one of the following three symptoms lasting for at least one day and no other obvious reason for the symptom is known.

1. Sore throat.
2. Cough (chronic coughing, e.g. due to smoking, will not be reported except if acutely worsening)
3. Nasal symptoms: (discharge, obstruction, tickling, sneezing). Previously known hay fever or other allergic symptoms will not be recorded except if acutely worsening)

Following additional symptoms may or may not be associated with an acute respiratory infection: headache, muscular ache, malaise, conjunctivitis, fever, breathing difficulty, or chest pain.

Gastrointestinal infection

A person will be defined to have an acute gastrointestinal tract infection if she/he is having at least one of the following two symptoms for at least one day and no other obvious reason for the symptom is known.

1. One or more rounds of vomiting (travelling malaise, or pregnancy associated malaise not to be recorded)
2. Looser than normal stools at least three times within 24 hours.

A gastrointestinal infection may or may not be associated with malaise, abdominal pain, or fever.

References:

1. Savolainen-Kopra C, Haapakoski J, Peltola PA, Ziegler T, Korpela T, Anttila P, et al. STOPFLU: is it possible to reduce the number of days off in office work by improved hand-hygiene? Trials. 2010;11:69. PubMed PMID: 20525328. Epub 2010/06/08. eng.

2. Savolainen-Kopra C, Haapakoski J, Peltola PA, Ziegler T, Korpela T, Anttila P, et al. Hand washing with soap and water together with behavioural recommendations prevents infections in common work environment: an open cluster-randomized trial. Trials. 2012;13:10. PubMed PMID: 22243622. Epub 2012/01/17. eng.
