# Supplementary material for: Development of a prognostic model based on demographic, environmental and lifestyle information for predicting incidences of symptomatic respiratory or gastrointestinal infection in adult office workers
Source: Trials. 2016 Nov 16;17:545. doi: 10.1186/s13063-016-1668-7 (PMC5112653; doi:10.1186/s13063-016-1668-7)
Supplement: Additional file 3: — Additional descriptive and statistical analyses. Time distribution of enrollment of study participants, frequency of various monitoring periods, and reanalysis of potential significant predicting variables using a dataset with participants reporting data for 50 weeks or more. (PDF 237 kb) [file 13063_2016_1668_MOESM3_ESM.pdf]

Additional file 3. Descriptive and statistical analyses. Time distribution of enrolment of study participants, frequency of various monitoring periods, and re-analysis of potential significant predicting variables using a dataset with participants reporting data for 50 weeks or more.

Distribution of enrolment dates

Onset of the intervention was scheduled for February 2009 but because of unavoidable organizational changes in one major participating corporation, part of the clusters started the study in March 2009. As shown in Fig.1., enrolments occurring later were distributed through the remaining 14

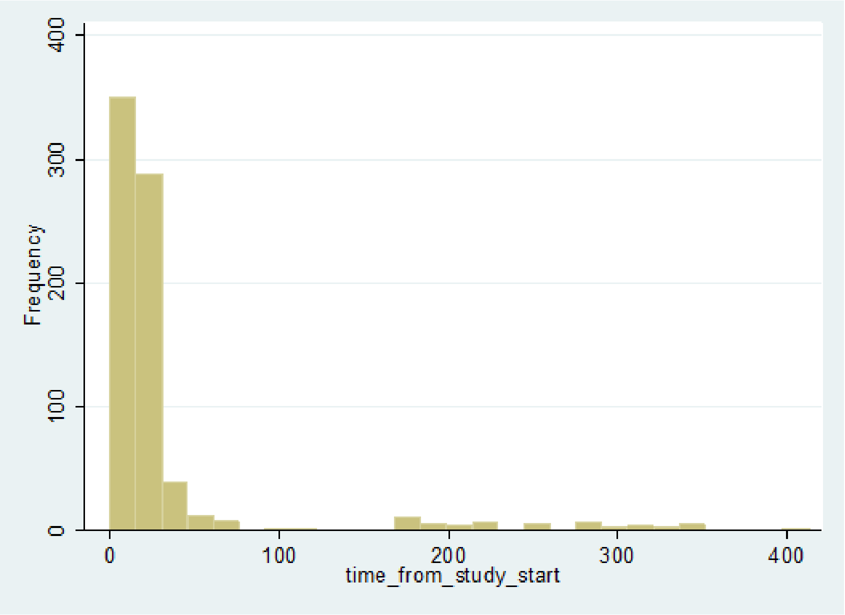

Fig.1. Distribution of enrolment days of the 717 . The time is in days.

months of the trial according to recruitment of new staff members into the participating working teams (clusters).

Variation of reporting duration by individual participants

In principal, the participants were supposed to send the weekly reports throughout the duration of the trial, maximally for 16 months. However, according to the protocol weeks on holiday were exempted even though reporting was also allowed during the holidays. It was clear at the onset of the trial that there will be changes in the membership of the study clusters during the course of the trial. In order to maintain the number of reporting participants as high as possible it was decided that new recruits in the teams will be offered a possibility to participate in the trial. As a consequence, the total time individual participants sent the weekly reports on exposures to and symptoms of respiratory tract (RTI) or gastrointestinal tract infections (GTI) was variable. Distribution of the duration of participation is shown in Fig.2.

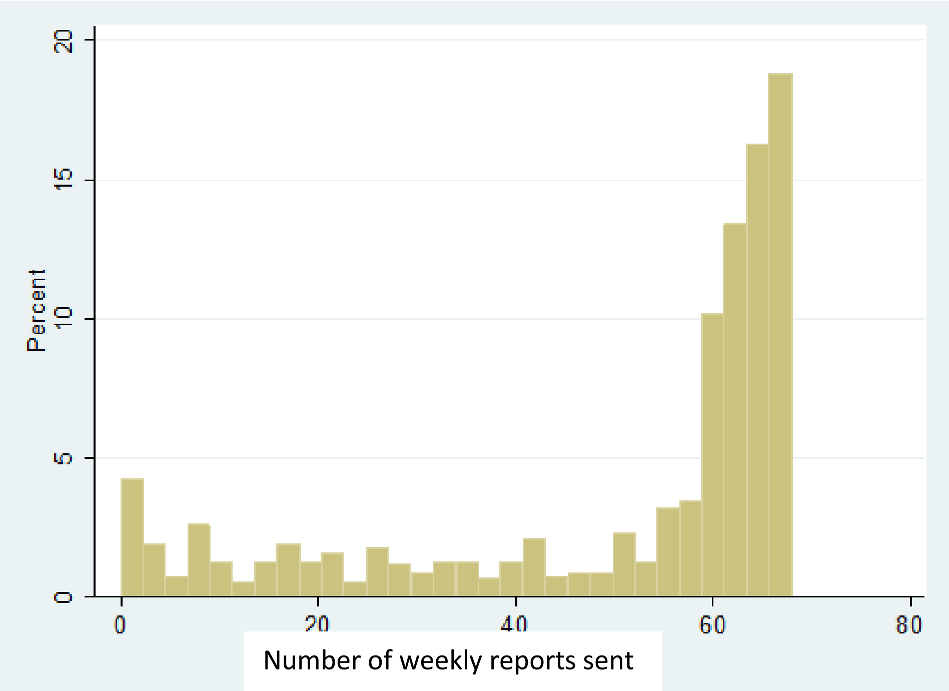

Fig.2. Variation of reporting duration. The height of the columns indicates the percentage of participants sending the indicated number of reports.

Reanalysis of the data by excluding persons shorter than 50 week duration

Mixed effect negative binomial regression model (1,2) with backward variable selection using AIC criterion was used to identify statistically significant variables and to calculate the corresponding incidence rate ratios (IRR). The values calculated for the 50 weeks or more reporting subpopulation differed little from those of the total population in the case of RTI incidence (Table 1).

Table 1. Variable-specific RTI incidence rate ratios in participants with ≥50 follow-up weeks

variable	Participants reporting for ≥50 weeks			All participants		
	IRR	CI	p	IRR	CI	p
Age	0.975	0.968, 0.983	0.000	0.975	0.968, 0.981	0.000
Outside-home day-care	1.229	0.977, 1.544	0.078	1.175	0.965, 1.431	0.109
Regular use of public transport	1.233	1.055, 1.442	0.009	1.199	1.041, 1.379	0.012
Chronic cardiovascular or respiratory disease	1.249	0.994, 1.568	0.056	1.226	0.999, 1.503	0.050
Influenza vaccination in 2008	1.327	1.093, 1.611	0.004	1.328	1.112, 1.587	0.002

RTI, respiratory tract infection; IRR, incidence rate ratio; CI, 95% confidence interval

In the case of GTI, the variable specific IRRs changed little but most likely because of the smaller total number of events, the significance of the difference from 1.0 was not confirmed (Table 2).

Table 2. Variable-specific GTI incidence rate ratios in participants with ≥50 follow-up weeks.

Variable	Participants reporting for ≥50 weeks			All participants		
	IRR	CI	p	IRR	CI	p
Age	0.989	0.977, 1.001	0.091	0.983	0.973, 0.994	0.003
Gender	0.820	0.605, 1.113	0.203	0,695	0.529, 0.911	0.009
Chronic cardiovascular or respiratory disease	1.314	0.893, 1.931	0.165	1.284	0.915, 1.804	0.149

GTI, gastrointestinal tract infection; IRR, incidence rate ratio; CI, 95% confidence interval
